# Clinical application of mask region-based convolutional neural network for the automatic detection and segmentation of abnormal liver density based on hepatocellular carcinoma computed tomography datasets

**DOI:** 10.1371/journal.pone.0255605

**Published:** 2021-08-10

**Authors:** Ching-Juei Yang, Chien-Kuo Wang, Yu-Hua Dean Fang, Jing-Yao Wang, Fong-Chin Su, Hong-Ming Tsai, Yih-Jyh Lin, Hung-Wen Tsai, Lee-Ren Yeh

**Affiliations:** 1 Department of Biomedical Engineering, National Cheng-Kung University, Tainan, Taiwan; 2 Division of Medical Radiology, E-Da Cancer Hospital, I-Shou University, Kaohsiung, Taiwan; 3 E-Da Cancer Hospital, Hepatobiliary and Pancreatic Cancer Collaborative Oncology Group, Kaohsiung, Taiwan; 4 Department of Medical Imaging, National Cheng Kung University Hospital, College of Medicine, National Cheng Kung University, Tainan, Taiwan; 5 Department of Radiology, School of Medicine, University of Alabama at Birmingham, Birmingham, Alabama, United States of America; 6 Department of Biomedical Engineering & Medical Device Innovation Center, National Cheng Kung University, Tainan, Taiwan; 7 National Cheng Kung University Hospital, Liver Cancer Collaborative Oncology Group, Tainan, Taiwan; 8 Division of General and Transplantation Surgery, Department of Surgery, National Cheng Kung University Hospital, College of Medicine, National Cheng Kung University, Tainan, Taiwan; 9 Department of Pathology, National Cheng Kung University Hospital, College of Medicine, National Cheng Kung University, Tainan, Taiwan; 10 Department of Radiology, E-Da Hospital, I-Shou University, Kaohsiung, Taiwan; Taipei Medical University, TAIWAN

## Abstract

The aim of the study was to use a previously proposed mask region–based convolutional neural network (Mask R-CNN) for automatic abnormal liver density detection and segmentation based on hepatocellular carcinoma (HCC) computed tomography (CT) datasets from a radiological perspective. Training and testing datasets were acquired retrospectively from two hospitals of Taiwan. The training dataset contained 10,130 images of liver tumor densities of 11,258 regions of interest (ROIs). The positive testing dataset contained 1,833 images of liver tumor densities with 1,874 ROIs, and negative testing data comprised 20,283 images without abnormal densities in liver parenchyma. The Mask R-CNN was used to generate a medical model, and areas under the curve, true positive rates, false positive rates, and Dice coefficients were evaluated. For abnormal liver CT density detection, in each image, we identified the mean area under the curve, true positive rate, and false positive rate, which were 0.9490, 91.99%, and 13.68%, respectively. For segmentation ability, the highest mean Dice coefficient obtained was 0.8041. This study trained a Mask R-CNN on various HCC images to construct a medical model that serves as an auxiliary tool for alerting radiologists to abnormal CT density in liver scans; this model can simultaneously detect liver lesions and perform automatic instance segmentation.

## Introduction

Liver cancer was the seventh most commonly diagnosed cancer (4.7%) and the leading cause of cancer death (8.2%) globally in 2018, with approximately 841,080 new cases and 781,631 deaths that year [1]. Among liver cancers, HCC accounts for >80% of primary liver tumors and is estimated to be the fourth most common cause of death from cancer worldwide [2]. HCC rates are particularly high in eastern and southeastern Asia and Africa [2, 3]. In recent decades, HCC has attracted attention in the United States and Western Europe due to its major risk factors including nonalcoholic steatohepatitis, alcoholism, and hepatitis C [4]. It frequently occurs in middle-aged men [5], and it is a major burden in many countries [6].

The surveillance of liver lesions depends on imaging features. Aggressive periodic imaging surveillance of patients with high-risk factors and liver cirrhosis (including nodule sizes of <1 cm) is practiced widely [7, 8]. The early stages of HCC as defined by the Barcelona Clinic Liver Cancer system involve a single tumor or three nodules of <3 cm [8]. In addition, the essential criteria of HCC defined by the American Joint Committee on Cancer staging system are one or multiple tumors and the largest having a diameter exceeding 2 cm or 5 cm.

Patients with early-stage HCC can be treated with curative resection or liver transplantation [8, 9]. Transarterial chemoembolization and radiofrequency ablation are treatment options for intermediate-stage HCC [8–10]. For advanced-stage HCC, systemic therapy with sorafenib remains the best option [9].

### Pathophysiology and imaging features of HCC

The liver has two major vascular inflow systems: arterial and portal systems. When HCC develops, the vascular supply system shifts to the arterial system. As a result, the typical features of dynamic imaging of HCC are early arterial enhancement and portal venous- or delayed-phase washout. The typical and atypical enhancement patterns of HCC on computed tomography (CT) were 56.4% and 43.6%, respectively [11]. The tumor growth pathology is a spectrum rather than a clear cutoff characteristic. Moreover, pathological classifications are made on the basis of variable types [12, 13]. HCCs of different sizes may manifest in the different phases of CT images as blurred margins, homogeneous enhancement, heterogeneous enhancement, poor enhancement, central scars or necrosis, or infiltrative type [14, 15]. Thus, the detection and segmentation of liver lesions are challenging.

### Human versus computer-aided detection of liver lesions

Abnormal density identification in each CT image is the crucial first step for radiologists followed by differential diagnosis in evaluating liver disease. The traditional standard imaging diagnosis for HCC consists of a semantic description of lesions on the basis of pathophysiology by radiologists trained in multiphasic dynamic imaging studies. The mean values of sensitivity for the combination of arterial and portal venous-phase imaging, arterial and delayed-phase imaging, and a combination of all three phases were 86.8%, 90.3%, and 93.8%, respectively [16]. The pooled per-lesion sensitivity and positive predictive value of contrast-enhanced CT were 73.6% and 85.8%, respectively [17]. The sensitivity for diagnosing HCC <2 cm by using contrast-enhanced CT was 52.9% [18]. The detection rates of HCCs <1 cm were between 17.4% and 20.6% [19], and small regions with abnormal density were frequently missed or not evident on CT at the initial diagnosis by humans.

Imaging feature identification and HCC analysis in dynamic imaging studies are complex [20, 21], with various sizes and morphologies observed in practical clinical situations. In the past 5 years, imaging feature analysis has rapidly developed [22, 23], and digital imaging features can be computed from regions of interest (ROIs). It is a new field but a promising technology for liver lesion diagnosis [24].

Computer-aided detection (CAD) involves the identification of specific digital imaging features of target lesions; this assists clinicians in reducing observational oversights [25, 26]. Recently, convolutional neural networks (CNNs) have become mainstream for digital imaging analysis [27, 28]. A CNN is an advanced filter that transforms low-level imaging features into abstract high-level features. Some studies have used early CAD for liver tumor detection [29] and segmentation [30] and differentiation of liver masses on dynamic contrast medium-enhanced CT [31].

These CAD studies have revealed a segmentation quality for liver tumors similar to human performance (mean Dice coefficients of 0.69 vs. 0.72) but inferior to detection performance (recall: 63% vs. 92%) in the liver tumor segmentation test [32]. According to our limited knowledge, the best volume Dice score for liver tumor segmentation is 0.70 based on the Liver Tumor Segmentation Benchmark [33] in 2019. Until July 27, 2020, the optimal Dice value per patient was 0.7990 for three-dimensional (3D) volume segmentation of liver lesions on contrast-enhanced CT scans obtained from the Liver Tumor Segmentation Challenge (LiTs) with automatic segmentation algorithms (https://competitions.codalab.org/competitions/17094#results).

### Semantic segmentation using convolutional neural network

Many deep learning (DL) architectures for liver segmentation perform well on different datasets (e.g., LiTs, 3D Image Reconstruction for Comparison of Algorithm Database) or their own datasets with different window ranges.

The semantic segmentation architecture U-Net was first proposed in 2015 [34], with the 3D U-Net variant developed in 2016 [35]. Christ et al. [36] used a two-step modified form of U-Net [34] to perform liver lesion segmentation and reported a Dice value greater than 0.94. Xiao developed a deep convolutional neural network [37] combining the features of U-Net [34] and ResNet [38] and achieved an average Dice value of 0.67. The H-DenseUNet [39] demonstrated aa global Dice value of 0.965 for liver segmentation and 0.824 for liver lesion segmentation using a hybrid feature layer. AHCNet [40] demonstrated global Dice values of 0.959 for liver segmentation and 0.734 for tumor segmentation using a U-Net variant, attention mechanism, and skip connections. U^n^-Net, with an n-fold network architecture [41], resulted in a Dice value of 0.9638 for liver segmentation and 0.7369 for tumor segmentation. E^2^NET [42], a two-dimensional (2D) edge-enhanced network with a multiscale feature extraction backbone and a decoder similar to that of U-Net [34], yielded global Dice values of 0.968 and 0.829 for liver and tumor segmentation, respectively, on the LiTs dataset.

U-Net [34] is a semantic segmentation architecture that performs segmentation of classes without instance segmentation, which distinguishes objects of the same class. The original U-Net was tested on cell tracking. For more complex medical images, such as those of liver tumors, the careful design of liver-oriented CNN layers and the reconciliation of image features from different scales to U-Net are crucial.

### Mask region-based convolutional neural network

Girshick et al. proposed a region-based CNN (R-CNN) in 2013–2014 that consists of four basic components: image input, region extraction proposals, computation of CNN features, and classification of regions [43]. The R-CNN can converge the diverse morphology, size, color, density, and texture characteristics as well as the visualization of domain knowledge into a digital informatics system for further transferring applications and engagements to other experts. On the basis of this structure, fast [44], faster [45], and Mask R-CNN by He et al. [46] were developed and exhibited high efficiency for simultaneous object detection and instant segmentation regarding the relationship between object imaging features and background. For Mask R-CNN, ROI Align was proposed to solve the problem of misalignment. Thus, the object size is no longer a problem if the target item provides sufficient pixel information. As a result, the inference ability of Mask R-CNN for images with complicated pixel information of HCC is unknown.

In this study, we used datasets from two hospitals in Taiwan rather than the open-source dataset. The assessment was conducted from radiologists’ viewpoint in addition to engineering considerations.

To implement Mask R-CNN, this study used Google TensorFlow, the pretrained Microsoft Common Objects in Context model, and CT datasets to develop an inference model for abnormal liver density detection and segmentation simultaneously.

In this study, we trained the model based on the CT images of HCC obtained from one institute and further tested this model on the dataset obtained from the second institute.

## Materials and methods

### Collection of clinical training and testing datasets

**Fig 1:**
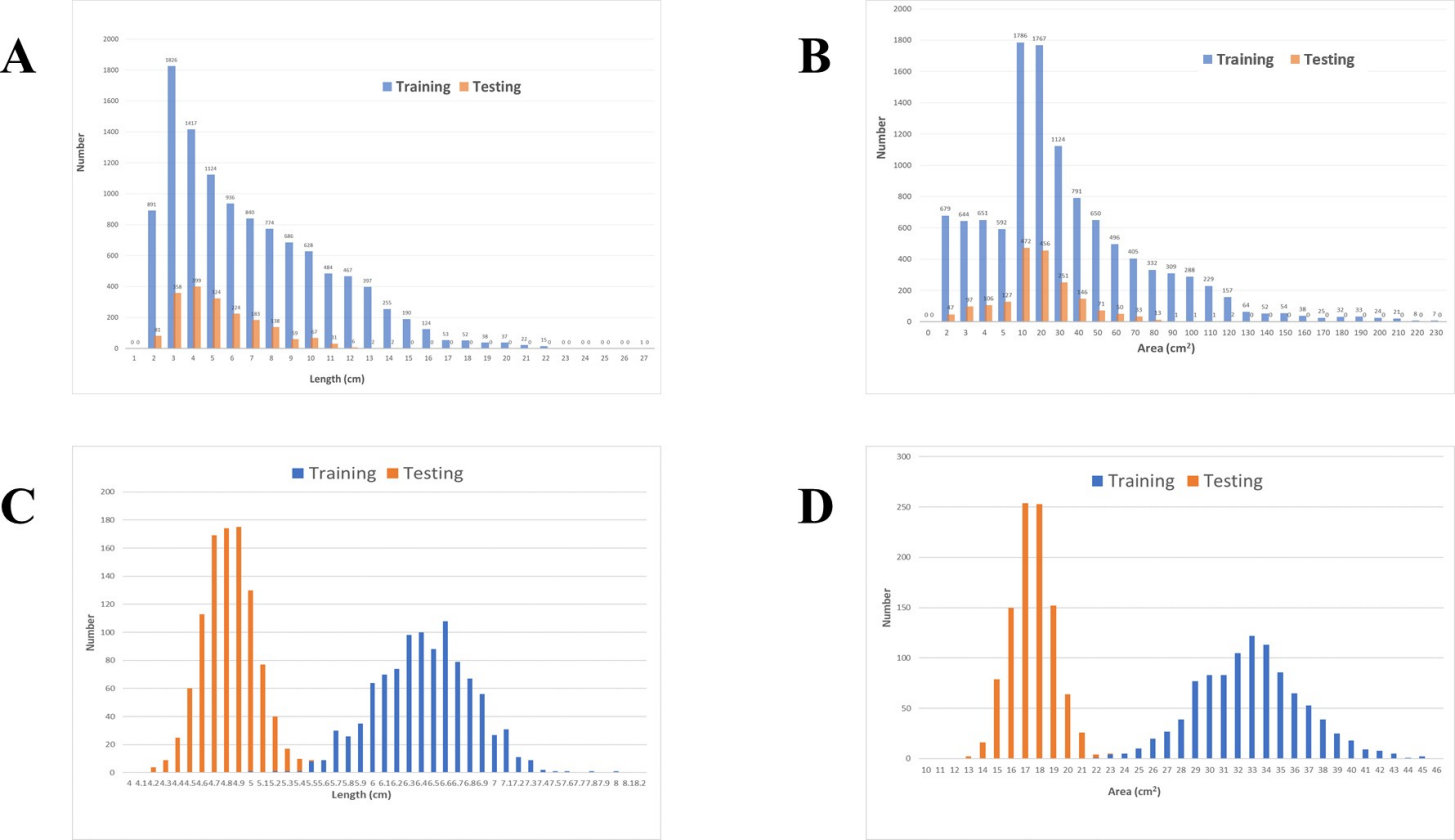
(A) Distribution of LDs of Abnormal Liver Density. (B) Distribution of areas of abnormal liver density. (C) Distribution of LDs after bootstrap resampling with a mean value of 100 samples repeated 1,000 times (*r* = 0.9980, *p* < 0.0001). (D) Distribution of areas after bootstrap resampling with a mean value of 100 samples repeated 1,000 times (*r* = 0.9988, *p* < 0.0001).

This retrospective study was approved by the institutional review boards of E-Da Hospital and National Cheng Kung University Hospital (EMRP-106-041/NCKUEDA10614; A-ER-107-023), which also waived the requirement of informed consent. HCC CT data from 2012 to 2017 were retrospectively obtained from the cancer databanks of two hospitals in Taiwan (Table 1 and Fig 1); all data were fully anonymized.

**Table 1:** Properties of training and testing data.

Characteristic	Training set (training: validation = 9:1 randomly distributed)	Positive test set	Negative test set
**Data source**	Hospital 1	Hospital 2	Hospital 2
**Number of patients**	394	214	123
**Male/ Female**	303/ 91	154/ 60	90/ 33
**Age (mean, SD)**	(64.9, 11.1)	(62.6, 11.2)	(59.9. 9.7)
**Dynamic/ Non-dynamic CT**	376/ 18	208/ 6	117/ 6
**Number of liver CT images**	10130	1833	20283
**Number of tumor ROIs**	11258	1874	-
**Number of hyperdense ROIs**	3078	334	-
**Number of hypodense ROIs**	6544	1313	-
**Number of heterogeneous density ROIs**	1636	227	-
**LD of GT tumor ROI (mean, SD) (cm)**	(6.37, 3.97)	(4.75, 2.16)	-
**Area of GT tumor ROI (mean, SD) (cm** ^**2**^ **)**	(32.08, 37.29)	(17.03, 15.83)	-

LD: longest diameter.

ROI: region of interest.

GT: ground truth.

SD: standard deviation.

The CT scanning parameters were 120 kVp with automatic exposure control depending on the CT machine. The time settings of the high-pressure syringe dynamic CT scans were 15–20 sec for the early arterial phase, 45 sec for the late arterial phase, 80 sec for the portal venous phase, and 4 min for the equilibrium delay phase, with an injection speed of 2.0–3.5 mL/sec. For nondynamic CT, the contrast medium was manually injected into the venous system of the upper extremity by a qualified nurse, and an immediate scan was conducted after the contrast medium was pushed out for the post-contrast-medium phase.

All images were acquired using 5-mm-thick transverse planes spaced at 5 mm in addition to coronal or sagittal reconstructed planes. All training images comprised only the field of view of the liver, and images of patients with severe fatty liver [47], hemochromatosis, and severe distortion of the liver parenchyma after transarterial chemoembolization, radiofrequency ablation, or partial resection were removed.

The images were converted from DICOM to 8-bit PNG format with a ratio of 512 × 512 pixels corresponding to 35 × 35 cm^2^ in 2D images under a window width of 450 HU and window level of 45 HU.

### Determination of ROI area and longest diameter

Because of varying ROI morphologies, the study used the fitEllipse and contourArea functions of the Open Source Computer Vision Library to automatically measure longest diameters (LDs) and areas through contour delineation of the lesions.

Finally, we acquired 10,130 images containing liver tumor densities of 11,258 ROIs (LD: 6.37 ± 3.97 cm; area: 32.08 ± 37.29 cm^2^) obtained from 394 CT images (dynamic CT to nondynamic CT ratio was 376:18) of 394 patients (age: 64.92 ± 11.14 years; male to female ratio was 303:91) as the training dataset from the first hospital (Table 1). In clinical viewpoints, the arbitrary 2D imaging features of one tumor may be similar with those of another tumor. For the maximal acquisition of abnormal imaging features, each 2D image was treated independently and randomly distributed into the training and validation datasets at a ratio of 9:1. Moreover, in the second hospital, 1,833 images containing liver tumors with 1,874 ROIs (LD: 4.75 ± 2.16 cm; area: 17.03 ± 15.83 cm^2^) obtained from 214 CT images (dynamic CT to nondynamic CT ratio was 208:6) of 214 patients (age: 62.59 ± 11.24 years; male to female ratio was 154:60) were regarded as positive testing data, and 20,283 CT images obtained from 123 CT images (dynamic CT to nondynamic CT ratio was 117:6) of 123 patients (age: 59.96 ± 9.76 years; male to female ratio was 90:33) with liver fields of view without abnormal densities in the liver parenchyma were regarded as negative testing data (Table 1). Fig 1A and 1B show the LD and area distribution obtained from the two hospitals. Fig 1C and 1D show the correlation values of LD and area distribution.

### Positive ground truth ROI

**Fig 2:**
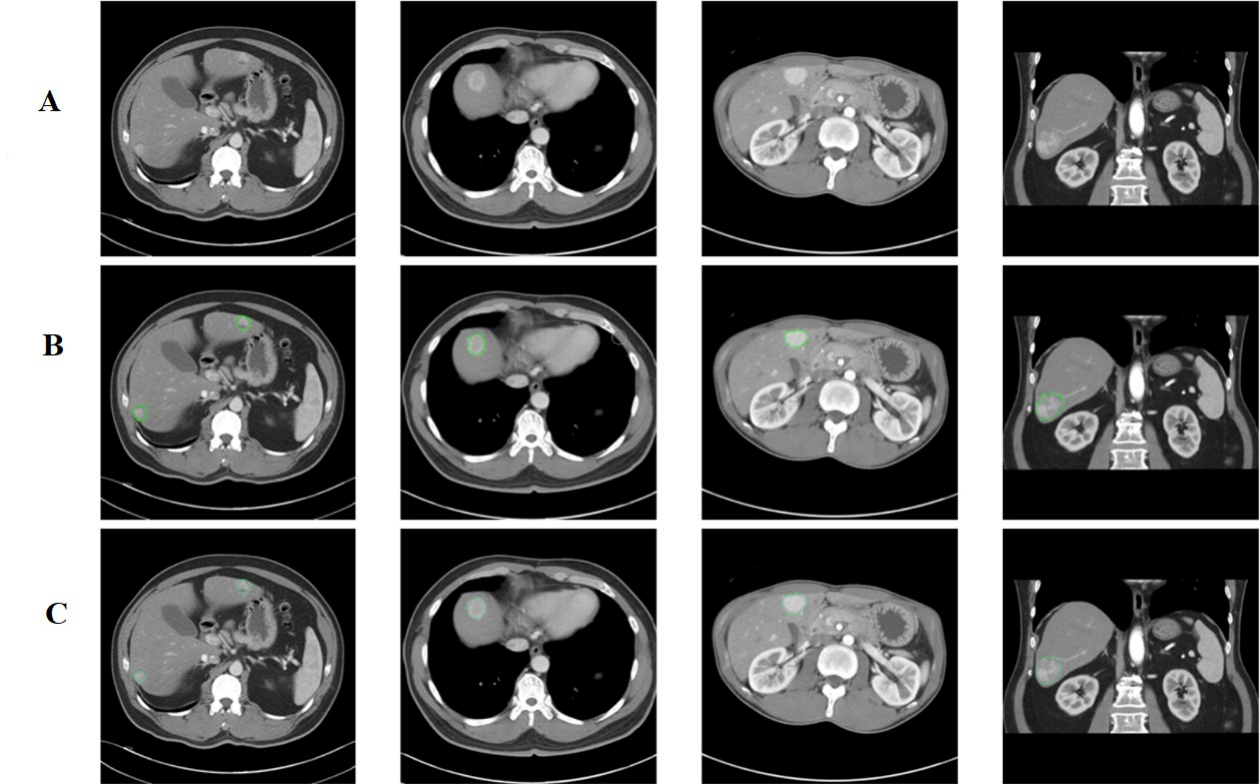
(A) Abnormal Hyperdensities Without Labeling ROI. (B) Hyperdensities with hand-marked ROIs. (C) Hyperdensities with AI-predicted ROIs.

**Fig 3:**
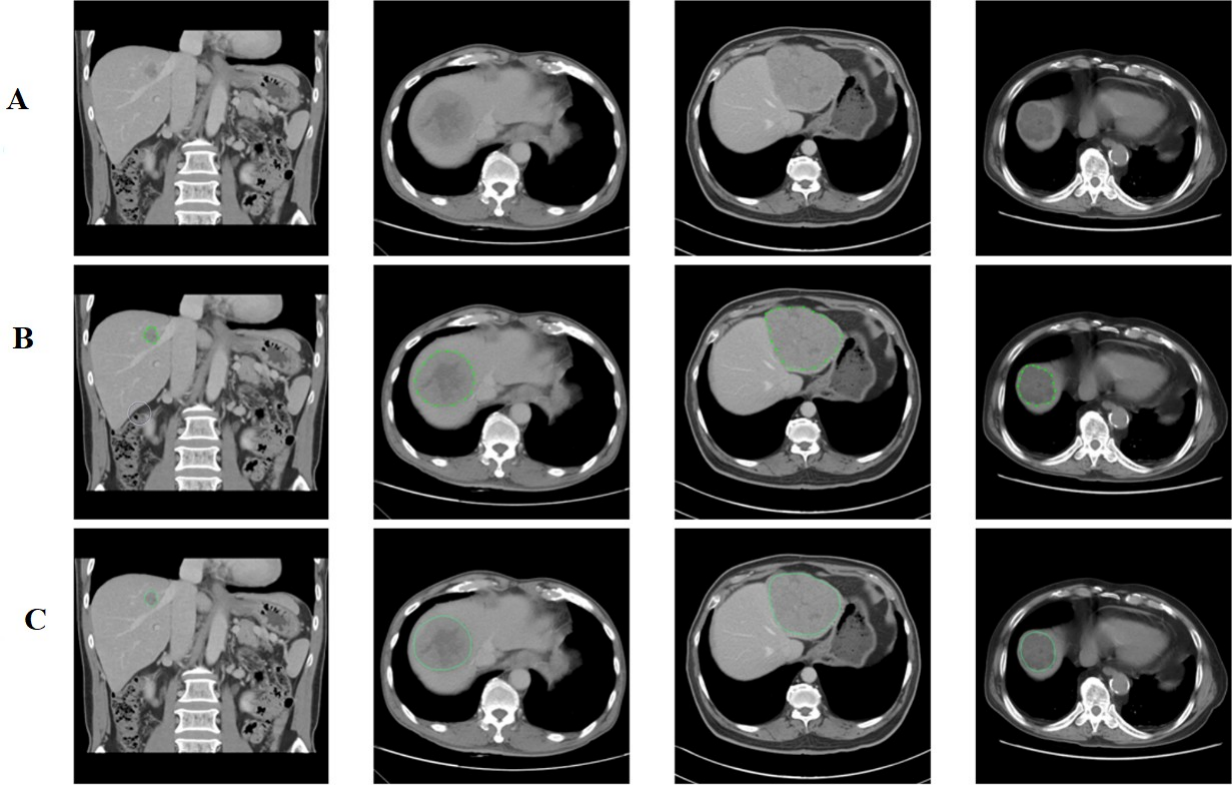
(A) Abnormal Hypodensities Without Labeling ROI. (B) Hypodensities with hand-marked ROIs. (C) Hypodensities with AI-predicted ROIs.

**Fig 4:**
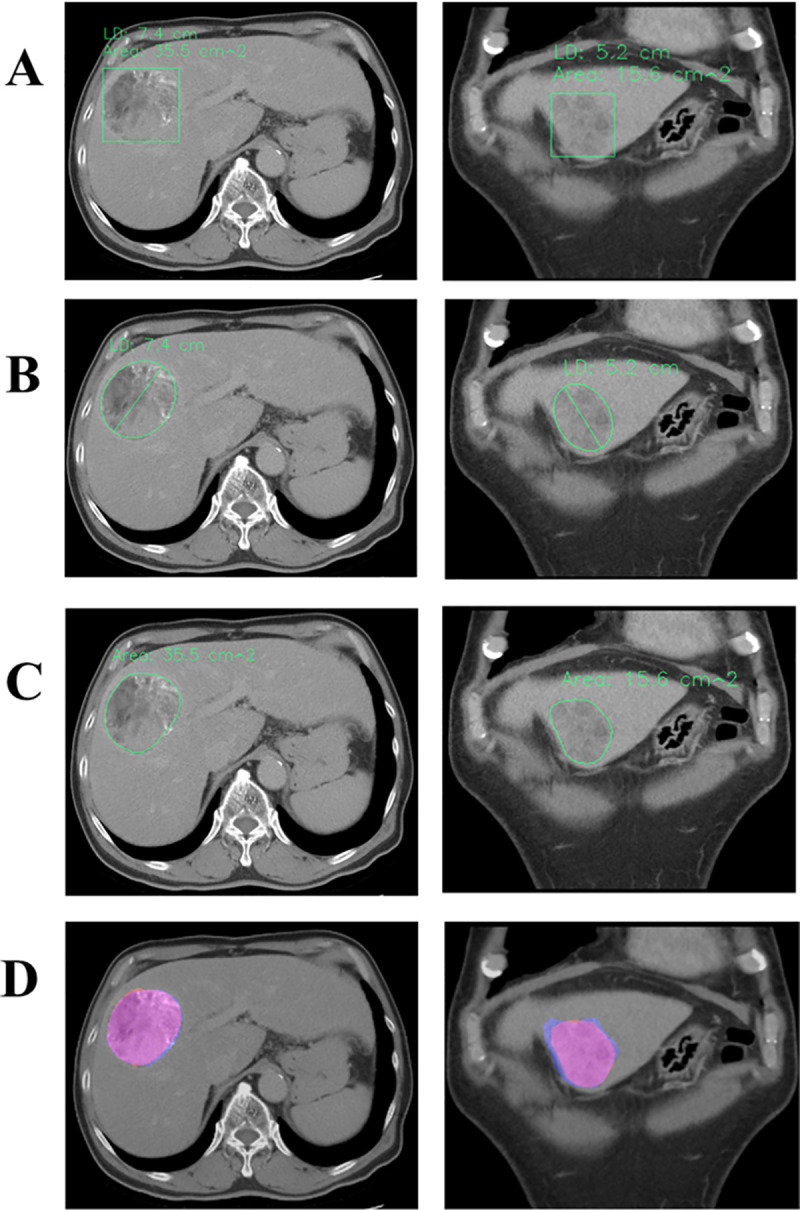
(A) AI-predicted Rectangle ROIs of Heterogeneous Densities with Concurrent Outputs of LD and Area. (B) AI-labeled LDs (7.4 and 5.2 cm) of abnormal densities through ellipse fitting. (C) AI-labeled areas of abnormal densities through contour delineation (area = 35.5 and 15.6 cm^2^). (D) Overlapping hand-drawn GT ROIs (light blue) and Mask R-CNN-predicted ROIs (pink) of abnormal densities (Dice coefficients = 0.9667 and 0.8922).

The positive ground truth (GT) tumors were verified against the cancer data bank document and original reporting document and further confirmed by a second radiologist. Abnormal liver densities were identified based on hyperdensity (Fig 2A) in the arterial phase, hypodensity (Fig 3A) in the noncontrast phase or washout patterns during either the portal venous or delayed phase, or heterogeneous density (Fig 4A–4C) [11].

### Negative GT dataset

The prevalence rate of incidental hepatobiliary findings based on chest CT screening was 6.1% [48]. The prevalence of small hepatic lesions discovered through CT in patients with cancer was 12.7% [49]. In addition to determining the positive GT, this study included the negative GT dataset without abnormal density in the liver parenchyma to mimic the larger number of normal images encountered in the daily practice of radiologists.

### Implementation of artificial intelligence inference

The experimental protocols of Protocol_mask_RCNN_CT_tensorflow were deposited in protocols.io with the following digital object identifier: dx.doi.org/10.17504/protocols.io.buusnwwe. We used public medical images from The Cancer Genome Atlas Liver Hepatocellular Carcinoma data collection (https://wiki.cancerimagingarchive.net/display/Public/TCGA-LIHC) [50] of The Cancer Imaging Archive [51] for preliminary program testing.

**Fig 5:**
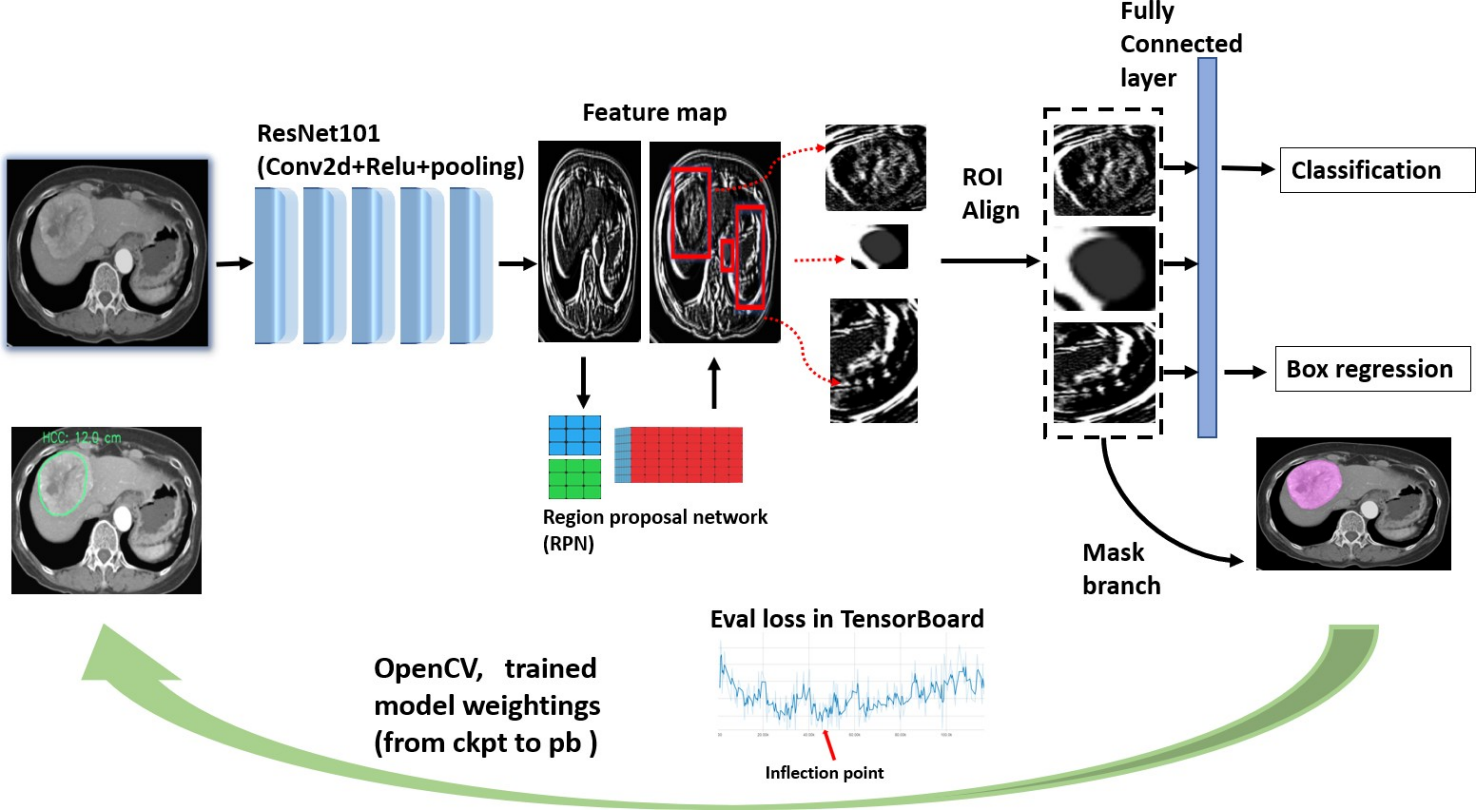
Diagram of mask R-CNN modeling and inference building.

Fig 5 depicts the end-to-end process of this inference model. Its backbone is ResNet101, which filters input images using 64 randomly generated filters in the first stage and constructs 101 layers. The residual learning of ResNet employs shortcut connections from one layer to subsequent layers that facilitate training of the deeper network [38]. We assumed that this backbone could capture more diverse features from HCC images. The design of the region proposal network incorporates the ROIs into a feature map following the RoI Align to avoid misalignment of image size and pixel information. In the final stage, the Mask R-CNN, employs bounding box regression and object classification using the neural networks. Thus, with sufficient pixel information, the two-stage architecture of the Mask R-CNN can perform accurate object detection and instance segmentation regardless of the size of object.

After building the training dataset, we used mask_R-CNN_resnet101_atrous_coco as the pretrained model. Microsoft’s Common Objects in Context is a large-scale image dataset designed for object detection and segmentation [52]. The training dataset comprises 10,130 images with a mixture of hypodensity, hyperdensity, and heterogeneous density (Table 1). The next step was the optimization of digital imaging features by using the Google TensorFlow open-source platform for machine learning on a NAVIDIA 2080 Ti GPU. All training images were input in batches (1,000–3,000 training images per batch) to the DL model and inspected using the TensorBoard web interface. The training endpoint was set as the inflection point of the validation data in the training dataset to avoid overfitting errors (Fig 5).

**Fig 6:**
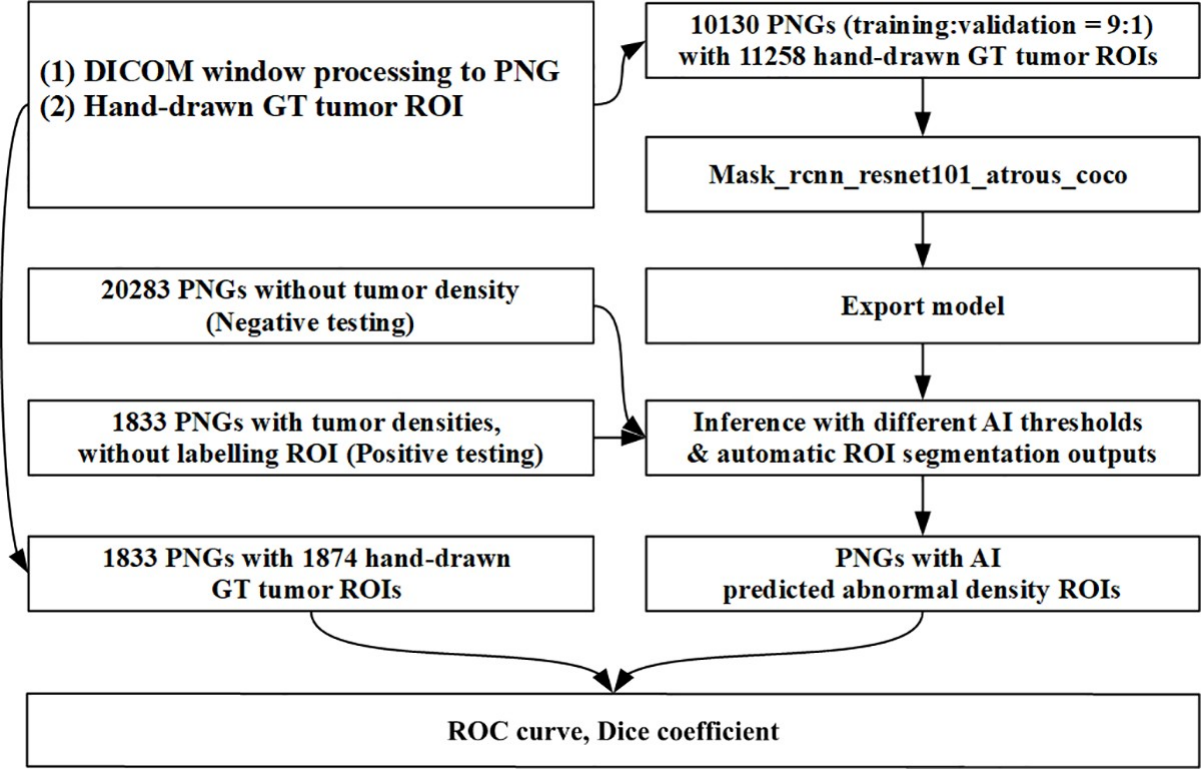
Process of mask R-CNN modeling and inference building.

Finally, the optimal model parameters were frozen and bridged to the Open Source Computer Vision Library to build the inference for further application, that is, testing with the dataset from the second hospital (Fig 6).

For this artificial intelligence (AI) inference, we designed two automatic detection output interfaces for use depending on user preferences: rectangle inference–predicted ROI (Fig 4A) and contour inference–predicted ROI (Fig 4C). Simultaneously, LD, area, and overlapping maps were revealed under contour delineation results (Fig 4B–4D).

### Statistical analysis

Because the training dataset was larger than the testing dataset in the right-skewed distribution (Fig 1A and 1B), a bootstrapping method [53] was employed (1,000 random samples of 100); an independent-samples *t* test [54] was used to compare the distribution of mean values by randomly resampling LD and area values from the training and testing datasets (Fig 1C and 1D).

The testing section was composed of two sets of mask thresholds (THs; 0.25, 0.5, and 0.75) and confidence THs (0.99, 0.95, 0.9, 0.85, 0.8, 0.75, 0.7, 0.65, 0.6, 0.55, 0.5, 0.45, 0.4, 0.35, 0.3, 0.25, and 0.2) of 51 combinations for determining variations in detection sensitivity.

In medical imaging detection, the most essential aspect of lesion detection is alerts of any suspicious abnormal density in the image, regardless of the lesion size. Thus, true positive was defined as any pixel intersection of the positive GT ROI and inference-predicted contour ROI larger than zero pixels. Furthermore, the Dice coefficient was determined through comparing the GT ROI and inference-predicted contour ROI for segmentation ability.


Dicecoefficient=2|GT_ROI∩Predicted_ROI|/(GT_ROI+Predicted_ROI) (1)

If the contour inference detected any pixel in the group of negative testing images, the result was regarded as a false positive.

The area under the curve (AUC), true positive rate (TPR), false positive rate (FPR), and Dice coefficient were used to evaluate liver lesion detection and segmentation ability.

## Results

The LD correlation and area correlation values between training and testing datasets were 0.9980 and 0.9988, respectively, and the *p* value of both was <0.0001 (Fig 1C and 1D). During training, in TensorFlow, the validation dataset at the inflection point revealed a loss value of 1.181.

Detection evaluation was assumed for 3 and 17 types of mask THs and confidence THs, respectively, to tune different sensitivities of the AI system for lesion detection. The average processing time by a 2080 Ti GPU for a single image was 0.51 sec. Because making absolute cutoff margins for lesion and non-lesion areas in a single image is impractical, the pathological scale of tumors cannot be precisely revealed through CT imaging. We designed positive and negative testing datasets for evaluating detection ability. The testing group included 1,833 images with 1,874 lesion ROIs (positive testing dataset) and 20,283 images without abnormal lesion densities (negative testing dataset).

**Fig 7:**
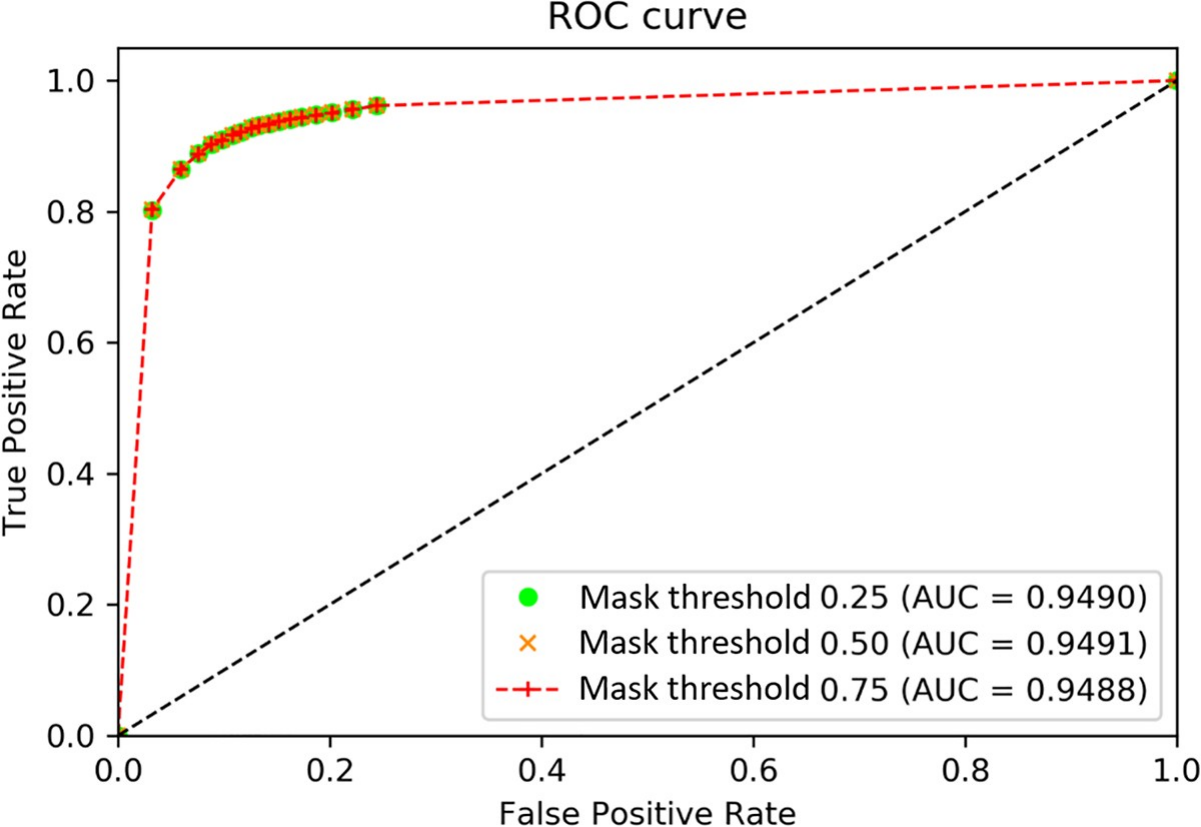
Receiver operating characteristic curve of lesion detection inference for all tumor densities.

This model obtained AUC values of 0.9490 (95% confidence interval [CI]: 0.9471–0.9508), 0.9491 (95% CI: 0.9472–0.9509), and 0.9488 (95% CI: 0.9469–0.9506) for the three mask THs of 0.25, 0.50, and 0.75, respectively (Fig 7), for a mixture of all three density conditions.

According to Fig 7, the optimal discrimination point is at the upper left corner of the receiver operating characteristic curve (mask TH = 0.50, confidence TH = 0.85). We further analyzed the performance of this inference for hyperdensity, hypodensity, and heterogeneous density under mask TH = 0.5, with different confidence THs.

**Fig 8:**
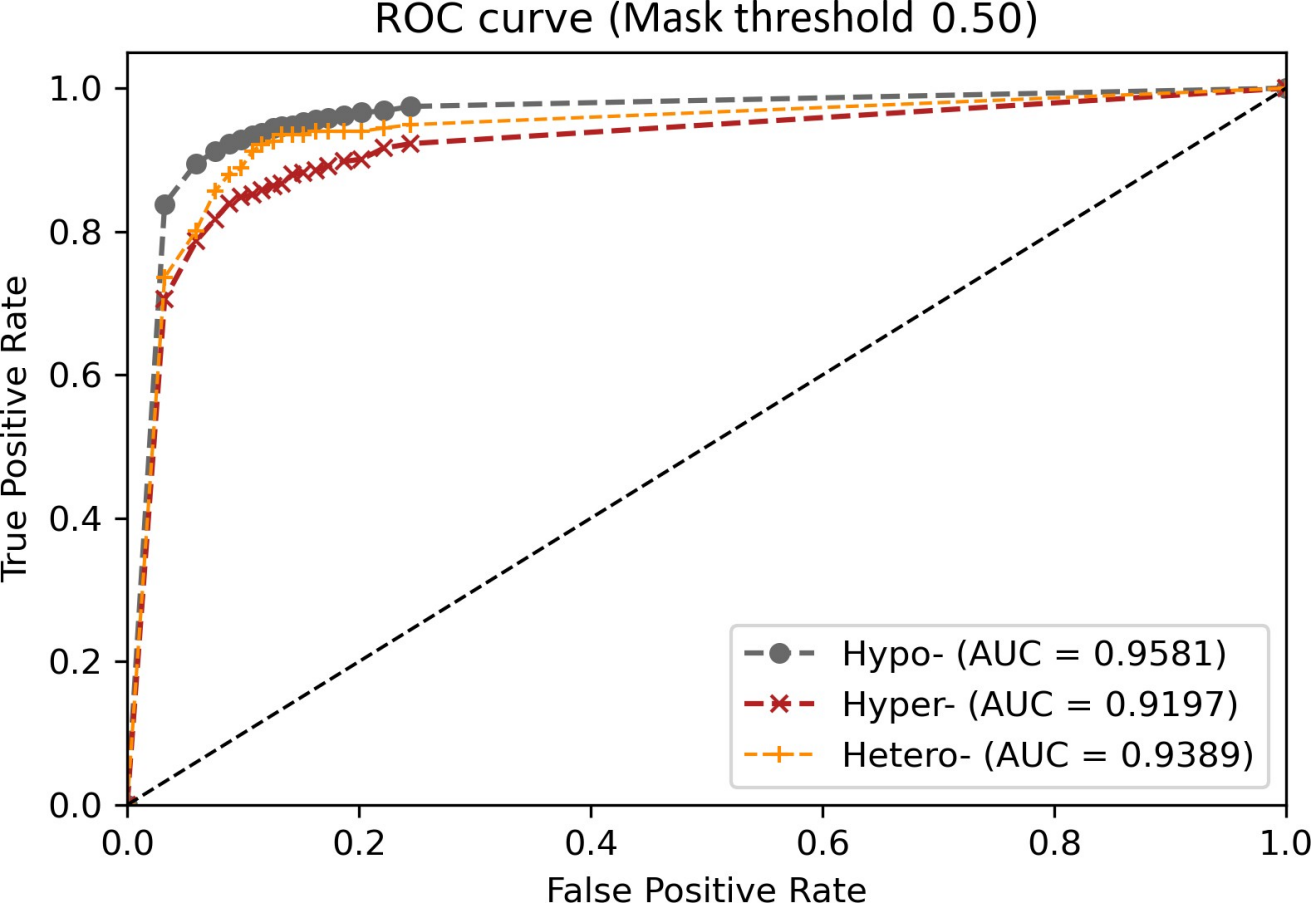
Receiver operating characteristic curve of lesion detection inference for different densities.

The optimal AUC value was 0.9581 (95% CI: 0.9561–0.9600) for hypodense lesions, followed by 0.9389 (95% CI: 0.9331–0.9446) for those of heterogeneous density and 0.9197 (95% CI: 0.9140–0.9253) for hyperdense lesions (Fig 8).

The mean AUC, TPR, and FPR were 0.9490, 91.99%, and 13.67%, respectively, for the objective detection of abnormal lesions with 51 TH combinations and a mixture of all three density conditions (Table 2).

**Table 2:** TPR (%), FPR (%), and dice coefficients of different confidence THs and mask THs.

Confidence TH	Mask TH = 0.25			Mask TH = 0.5			Mask TH = 0.75		
	FPR (%)	TPR (%)	Dice	FPR (%)	TPR (%)	Dice	FPR (%)	TPR (%)	Dice
**0.99**	3.24	80.25	0.7032	3.24	80.31	0.7100	3.24	80.31	0.6994
**0.95**	5.95	86.42	0.7545	5.95	86.47	0.7619	5.95	86.47	0.7499
**0.9**	7.6	88.87	0.7723	7.6	88.87	0.7796	7.6	88.82	0.7671
**0.85**	8.79	90.29	0.7838	8.79	90.29	0.7914	8.79	90.23	0.7787
**0.8**	9.84	90.94	0.7884	9.84	90.94	0.7961	9.84	90.89	0.7834
**0.75**	10.81	91.71	0.7944	10.81	91.71	0.8021	10.81	91.65	0.7892
**0.7**	11.64	92.14	0.7975	11.64	92.14	0.8053	11.64	92.09	0.7922
**0.65**	12.58	92.8	0.8030	12.58	92.8	0.8108	12.58	92.74	0.7975
**0.6**	13.37	93.13	0.8057	13.37	93.13	0.8135	13.37	93.07	0.8001
**0.55**	14.27	93.4	0.8076	14.27	93.4	0.8155	14.27	93.34	0.8021
**0.5**	15.18	93.78	0.8103	15.18	93.78	0.8182	15.18	93.73	0.8050
**0.45**	16.3	94.16	0.8131	16.3	94.16	0.8211	16.3	94.11	0.8078
**0.4**	17.38	94.44	0.8144	17.38	94.44	0.8225	17.38	94.38	0.8092
**0.35**	18.73	94.76	0.8173	18.73	94.76	0.8255	18.73	94.71	0.8123
**0.3**	20.21	95.14	0.8211	20.21	95.14	0.8294	20.21	95.09	0.8161
**0.25**	22.15	95.64	0.8236	22.15	95.64	0.8319	22.15	95.58	0.8187
**0.2**	24.47	96.24	0.8268	24.47	96.24	0.8355	24.47	96.18	0.8225
**mean**	13.68	92.01	0.7962	13.68	92.01	0.8041	13.68	91.96	0.7912
**SD**	5.8	3.95	0.0306	5.8	3.93	0.0310	5.8	3.92	0.0303

TH: threshold.

SD: standard deviation.

For the true positive detection of lesions, automatic CAD segmentation was performed. This inference attained mean Dice coefficients of 0.7962 (standard deviation [SD] = 0.0306), 0.8041 (SD = 0.0310), and 0.7912 (SD = 0.0303) for the three mask THs (Table 2). The segmentation ability achieved a mean Dice coefficient of 0.7971 for 51 TH condition combinations, with the best coefficient being 0.8041 under a mask TH of 0.5 and 0.8355 under a mask TH of 0.5 and confidence TH of 0.2. This inference exhibited acceptable detection and segmentation abilities.

**Fig 9:**
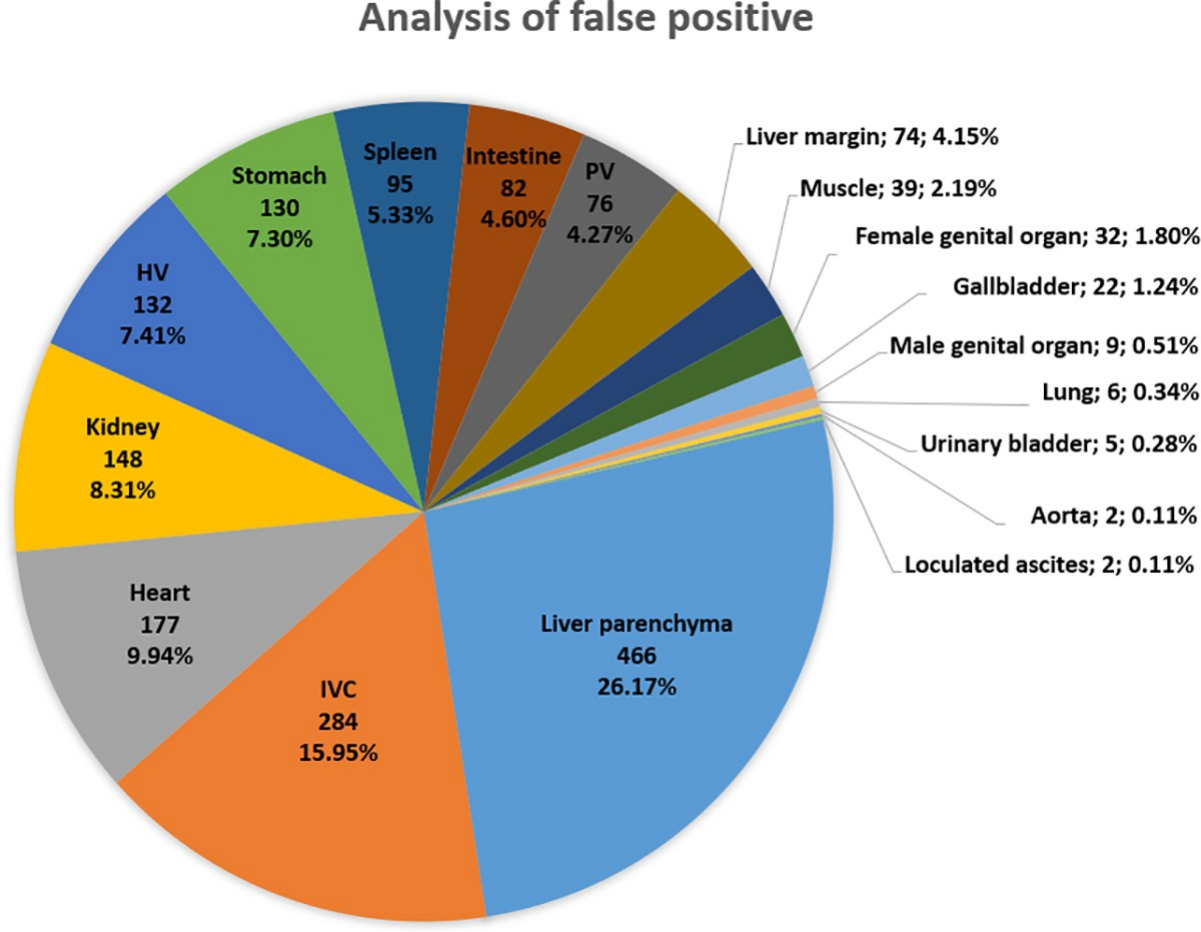
Analysis of 1781 false positive images. (IVC: inferior vena cava; HV: hepatic vein; PV: portal vein).

Uneven liver parenchyma (26.17%) accounted for the majority of false positives, followed by the inferior vena cava (15.95%) and other organs (Fig 9). These results provide essential information for the future improvement of this inference.

## Discussion

This study used the previously proposed Mask R-CNN to engineer and design of a method of data collection and model evaluation from a medical viewpoint. This 2D validation study performed training and testing on HCC datasets with different size distributions in an end-to-end manner.

The Mask-R-CNN extracts the feature map and simultaneously performs the RoI Align for objective detection and instance segmentation with variable sizes. It provided AUC values greater than 0.9 for different liver lesion densities for a 2D Dice value of approximately 0.80.

Setting an appropriate window is the first step in the process of image inspection for radiologists and in feature extraction based on the DL model.

The initial window setting of this study was within the abdominal window range for increased ease of transfer learning—the machine learning method whereby a pretrained model is reused as the starting training point for another similar task—for use with the abdominal window ranges of other radiologists.

Although the optimal theoretical TH setting was mask TH = 0.50 and confidence TH = 0.85, the end user can select different TH combinations of varying sensitivities for particular medical situations.

In the analysis of the false positives, we identified uneven liver parenchyma, such as that caused by fatty liver or severe liver cirrhosis, as the primary weakness of this model because we excluded such extreme liver parenchyma images from our training dataset. The majority of the other false positives were related to major vessels or hypervascular organs such as the inferior vena cava or heart. We surmised that these may present similar features to the imaging features of hypervascular tumors under a single DL model. In addition, this 2D model lacks 3D information (i.e., z-axis); thus, its recognition of 3D organ structures is suboptimal. In addition, the model was trained on single class, with limited objects in each image; 3D voxel multiclass training has greater requirements for hardware, graphical processing, and training time than its 2D counterpart. The balance between the available CAD system and the hardware requirements for DL training is a crucial concern in public health care worldwide.

In this model, we did not include other malignant tumors such as cholangiocarcinoma, metastasis, and other benign liver lesions such as liver cysts or hemangiomas for multiple object detection.

For long-term usage and development, this model can be used for purposes other than single object liver lesion AI training through transfer learning, thus avoiding data scarcity dilemmas to achieve the desired training endpoint.

Detailed region segmentation and sophisticated classifications of dynamic CT phases, other liver lesions, diverse liver parenchymal backgrounds, and normal variations of organ structures should all be considered for this DL model in the future.

## Conclusion

This study demonstrated the application of the Mask R-CNN for usage in clinical imaging to improve the accuracy of liver lesion detection based on CT imaging from a medical perspective. Our study revealed that this single DL model cannot replace the complex and subtle medical evaluations of radiologists, but it can reduce tedious labor.

In the future, an AI CAD should be developed to help radiologists with the challenging task of lesion detection and analysis of different domains.
